# Dose-effect relationship of different acupuncture courses on chronic insomnia disorder: study protocol for a randomized controlled trial

**DOI:** 10.3389/fpsyt.2023.1277133

**Published:** 2023-12-11

**Authors:** Fengya Zhu, Junqian Liu, Yuan Wang, Tingting Ma, Tianyu Wang, Bin Yang, Runqing Miao, Jie Wu

**Affiliations:** ^1^Acupuncture and Tuina School, Chengdu University of Traditional Chinese Medicine, Chengdu, China; ^2^Department of Preventive Treatment, Hospital of Chengdu University of Traditional Chinese Medicine, Chengdu, China

**Keywords:** acupuncture, chronic insomnia disorder, dose-effect relationship, randomized controlled trial, protocol

## Abstract

**Background:**

Chronic insomnia disorder (CID) is increasing in prevalence year by year, is long lasting, and potentially risky. Acupuncture has been widely used in the clinical management of this condition. However, there is still a lack of direct evidence on the dose-effect relationship between different acupuncture courses and clinical efficacy. To identify this relationship, we will design a randomized controlled trial to clarify the difference in efficacy of different acupuncture courses for CID.

**Methods and design:**

This is a prospective, parallel, single center randomized controlled trial. Two hundred and one participants with CID will be randomly divided into three groups (Group A, Group B, and Group C). The three groups will be given acupuncture therapy for 4, 6, and 8 weeks, three sessions per week, with at least 1 day between sessions. Follow-up will continue until the third month after the end of treatment. The primary outcome is the Insomnia Severity Index (ISI), and secondary outcomes include percentage of ISI < 8 points, the Pittsburgh Sleep Quality Index (PSQI), Self-Rating Anxiety Scale (SAS), Self-Rating Depression Scale (SDS), Fatigue Severity Scale (FSS), medication use, and safety.

**Discussion:**

This study is expected to provide direct evidence for the optimal treatment cycle of acupuncture for CID, as well as to facilitate health economic evaluation.

**Clinical trial registration:**

https://clinicaltrials.gov/, identifier [ChiCTR2300073711].

## 1 Introduction

Insomnia is a common clinical sleep disorder that is characterized by difficulty with falling asleep, maintaining sleep, waking early, decreased sleep quality, and reduced total sleep time, accompanied by daytime dysfunction (1), such as fatigue, decreased cognitive functioning, depression/pain or irritation, and physical discomfort (2). Epidemiological data show that the incidence of persistent insomnia in the past 4 years is 76.5% (3), and patients with insomnia symptoms in developed countries such as Europe and the United States is about 30–50% (4). Approximately 10% of the adult population suffers from an insomnia disorder and another 20% experiences occasional insomnia symptoms (5). During the COVID-19 pandemic, the prevalence of insomnia is even higher (6). Insomnia is often a chronic condition, with symptoms persisting in 86% of patients 1 year after diagnosis and in 59% of patients for more than 5 years (7). CID leads to a number of consequences, such as reduced social productivity, substantial work costs and increased health care costs (8, 9), totaling more than $100 billion annually in the USA alone (10). It is evident that CID places a heavy burden on both patients and healthcare systems worldwide.

The goals of treating CID are to improve the quantity and quality of sleep, reduce the distress and anxiety associated with poor sleep, and improve daytime functioning (11). Currently, the main treatments for insomnia are cognitive behavioral therapy (CBT), hypnotic drugs, and traditional Chinese medicine (TCM) (12). However, even though CBT is recommended as a first-line, non-pharmacological therapy by international guidelines (13), it has resulted in poor patient compliance due to the different qualifications of doctors, cumbersome content, and high cost. Hypnotic drugs mainly include benzodiazepine receptor agonists, antihistamines, and other types of drugs, but there is a risk of abuse and addiction (14). Long-term use is prone to adverse effects such as drowsiness, weight gain, daytime fatigue, vertigo, delirium, ataxia, paraphilic amnesia, and sleepwalking (12), and is even significantly associated with the occurrence of serious adverse events such as traffic accidents and falls in the elderly (15, 16).

Acupuncture, as an important complementary and alternative therapy, has been inherited for thousands of years and is widely used in the treatment of chronic diseases. Clinical studies have shown that acupuncture can significantly prolong the sleep time of insomnia patients, reduce the number of sleep awakenings, improve the quality of sleep, relieve daytime fatigue and negative emotions such as anxiety and depression, and improve cognitive function (17–19). However, with the development of a large number of clinical studies, acupuncture intervention programs and acupuncture treatment courses are different. Related studies have mostly focused on the frequency of acupuncture interventions as 3 sessions per week and once every other day, and the treatment period is permanently set at 4–8 weeks (20–23). Several meta-analyses have evaluated the effect of different acupuncture courses on the clinical efficacy of insomnia. Yu suggested that the effect of acupuncture began to take effect in the first week, and the efficacy continued to increase during the 4-week treatment period (24). Kim et al. noted that insomnia patients had no significant effect from acupuncture in the first 2 weeks, though their symptoms showed significant improvement after 3 weeks (25). Zhao et al. showed that at least 12 sessions of acupuncture could significantly improve the quantity and quality of sleep in patients with primary insomnia (26). However, there is little direct evidence on the dose-effect relationship between different acupuncture courses on CID.

Under the general trend of pursuing precision and standardization in clinical medicine, clarifying the dose-effect relationship of acupuncture is not only closely related to clinical decision-making, but also influences the evaluation of health economics (27). Some chronic diseases require an adequate treatment course to achieve therapeutic efficacy (28). This study will attempt to clarify whether the clinical efficacy of acupuncture for CID is no longer significantly different after completing a certain course of acupuncture treatment? Or does the clinical efficacy become more significant as the course of treatment increases? Therefore, we will conduct a randomized controlled trial to evaluate the efficacy of different acupuncture courses for CID.

## 2 Objectives

To compare the dose-effect relationship of different acupuncture courses on CID.

## 3 Design and methods

### 3.1 Trial design and setting

**Figure 1:**
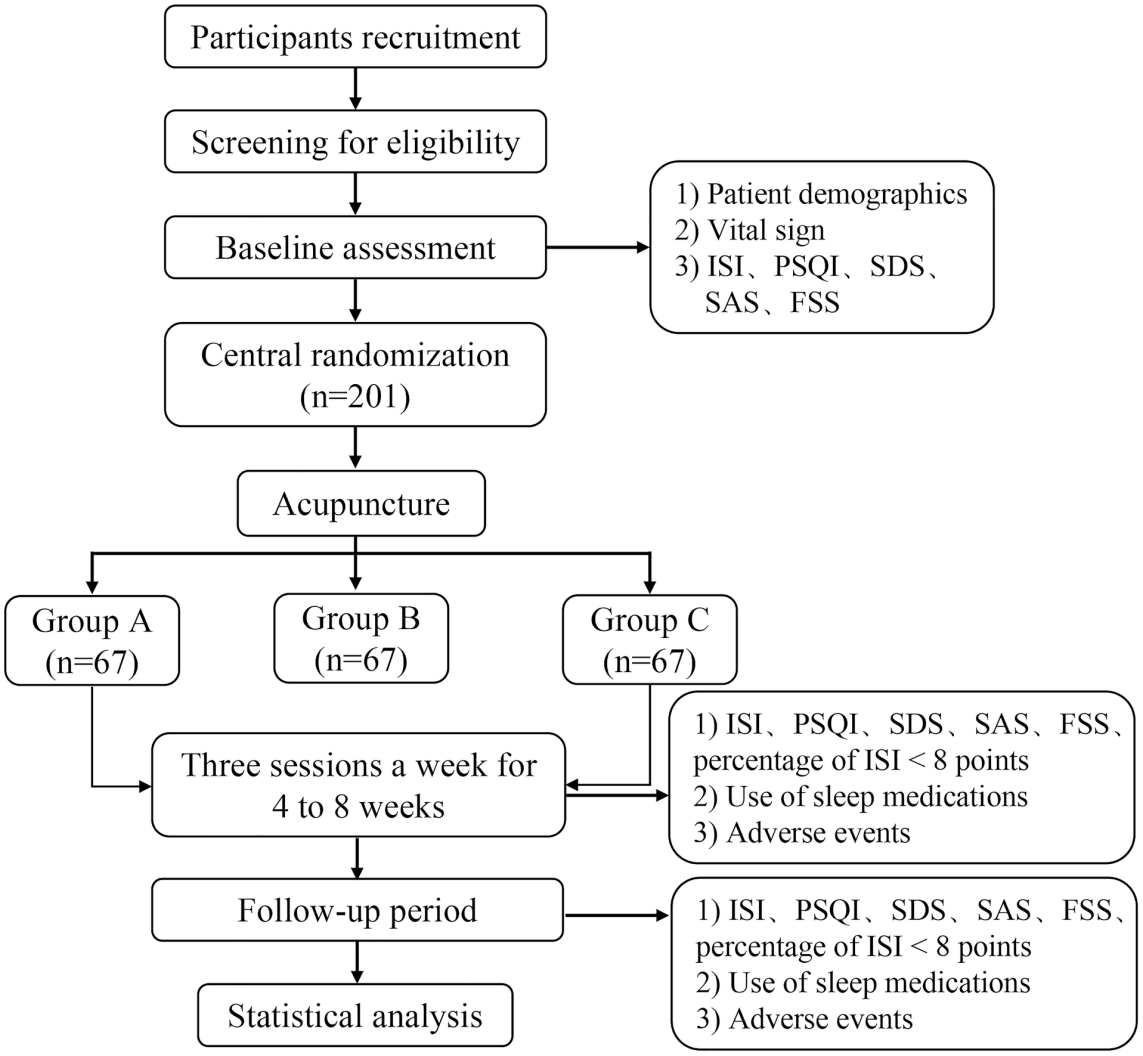
The flowchart of trial procedures.

This parallel, single center randomized controlled trial will be conducted from August 1, 2023, to February 28, 2025, at the Center for Preventive Medicine, Hospital of Chengdu, University of Traditional Chinese Medicine. We will strictly adhere to the Declaration of Helsinki and the Standards for Reporting Interventions in Clinical Trials of Acupuncture (STRICTA) guidelines (29). The Ethics Committee of Hospital of Chengdu University of Traditional Chinese Medicine has approved the protocol (2023KL-064) and registration was completed in the China Clinical Trial Registry (ChiCTR2300073711). Two hundred and one participants will be randomly assigned to three groups and receive different courses of acupuncture treatments. Each participant will be made aware of the purpose of the study and the randomization procedure. Acupuncture treatment, efficacy evaluation, and medical advice were all free of charge during the observation period. The flowchart of trial procedures is shown in Figure 1.

### 3.2 Participants

We plan to recruit participants through hospital outpatient clinics, WeChat e-posters, campuses, and communities. A researcher is responsible for screening eligible participants, and those successfully enrolled will be asked to voluntarily sign a written informed consent form (20,230,424, V2.0).

### 3.3 Eligibility criteria

Participants will need to meet the following inclusion criteria: (1) meet the diagnostic criteria for CID in the International Classification of Sleep Disorders, 3rd Edition (ICSD-3) (30); (2) Insomnia Severity Index (ISI) ≥ 10 score (31); (3) Gender is not limited, age 18–70 years old; (4) have not received any acupuncture interventions in the month prior to enrollment; (5) be able to communicate normally and actively cooperate with treatment; and (6) voluntarily enrolled and had signed an informed consent form.

Those who meet any of the following criteria will be excluded: (1) combined cardiovascular, cerebrovascular, neurological, digestive, endocrine system, and other serious diseases; (2) participants with severe depression [Hamilton Depression Scale (HAMD) > 24 score]; (3) severe neuropsychiatric disorders, such as cognitive impairment, lifelong bipolar disorder, any type of schizophrenia, paranoia, dementia, organic brain dysfunction, etc.; (4) those who abuse or rely heavily on psychoactive substances such as antidepressants, central stimulants, analgesics and sedatives, and alcohol; (5) secondary insomnia caused by other organic diseases; (6) pregnant or lactating women; (7) metal allergy; and (8) participants who are enrolling in other topics.

### 3.4 Randomization and blinding

Dynamic block randomization will be used to assign participants to three groups at a 1:1:1 ratio. The online central randomization system is undertaken by Chengdu CIMS Medical Technology Co. The generation of the randomized sequences will be completed by the designated investigator and participants will be given a unique number assigned by the system. Electronic data collection and data management systems will be set up by independent personnel.

The physicians responsible for the acupuncture treatments and participants in this study cannot be blinded. The data collectors, statistical analysts, and efficacy evaluators are unaware of the groupings.

### 3.5 Sample size

Sample size estimation was based on the change in the primary outcome ISI score. Based on the results of existing studies in the literature, the ISI score after 4 weeks of treatment was (10.5 ± 3.8) points (32), and we expected that after 6 and 8 weeks of treatment, the ISI score would decrease by 1 point and 2.5 points. With the test level set at *α* = 0.05 and the test efficacy at 1-β = 0.9, plus calculations based on the mean comparison formula of multiple independent samples yielded 58 cases in each group. Considering a 15% dropout rate, the three groups are evenly divided at 1:1:1, with 67 cases required in each group, for a total of 201 participants are needed for this trial.

Sample size estimation formula:


$$n=\frac{\psi^{2}\left(\sum_{j=1}^{k}s_{j}^{2}/k\right)}{\sum_{j=1}^{k}{\left({\bar{X}}_{j}-\bar{\bar{X}}\right)}^{2}/\left(k-1\right)}$$

### 3.6 Intervention

**Figure 2:**
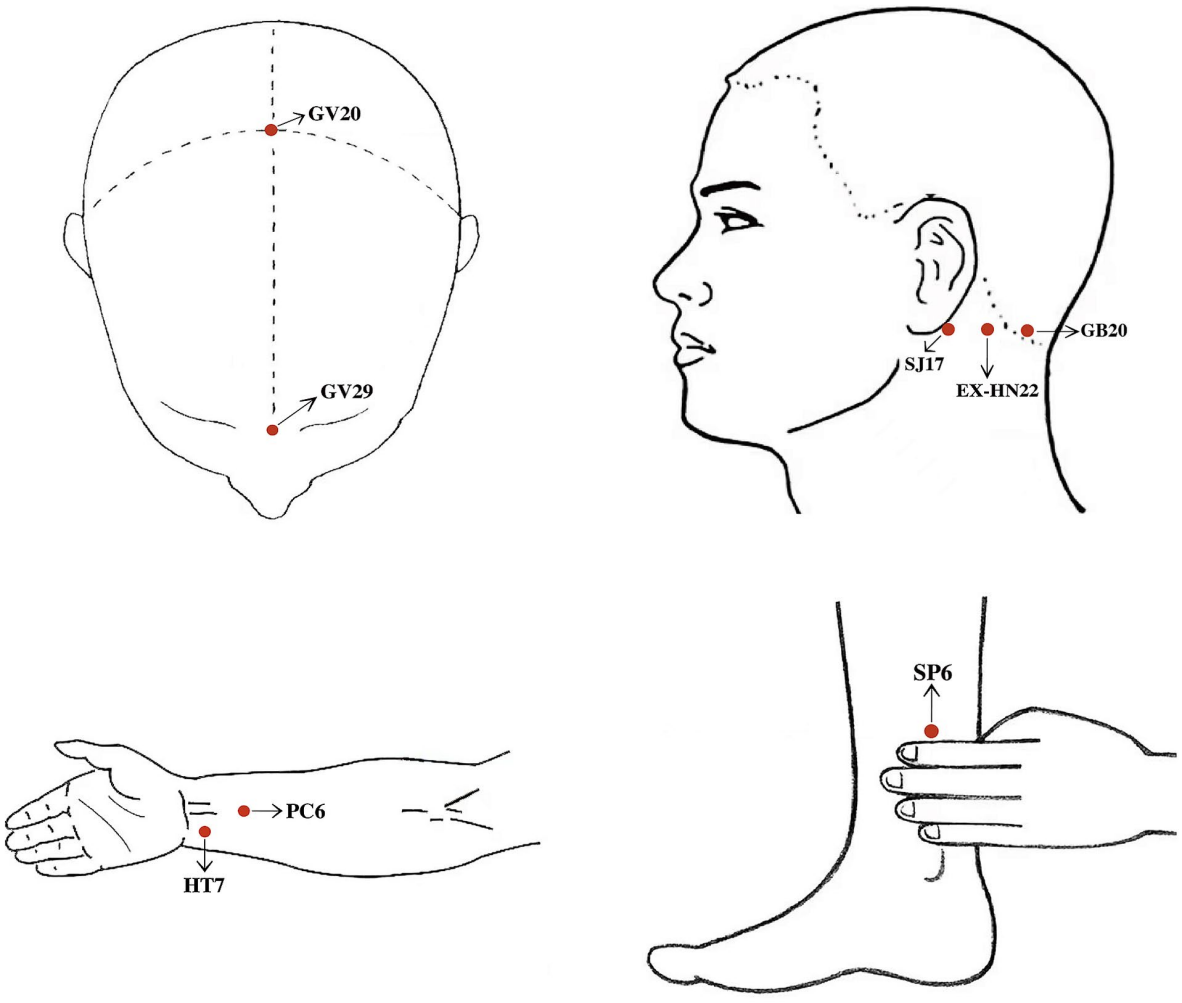
① Baihui (GV20): 5 cun superior to the anterior hairline, on the anterior midline of the head. ② Yintang (GV29): At the midpoint between the medial ends of eyebrows. ③ Anmian (EX-HN22): at the midpoint between Fengchi (GB20) and Yifeng (SJ17). ④ Shenmen (HT7): On the palmar ulnar end of the transverse crease of the wrist, and on the radial aspect of the tendon of the ulnar flexor m. of the wrist. ⑤ Neiguan (PC6): 2 cun above the transverse crease of the wrist, between the tendons of m. palmaris longus and m. flexor carpi radials. ⑥ Sanyinjiao (SP6): Posterior to the mesial border of the tibia and 3 cun above the tip of the medial malleolus.

Participants in the three groups receive the same acupuncture treatments three sessions a week with at least 1 day between the two treatments: Group A (12 sessions for 4 weeks), Group B (18 sessions for 6 weeks), and Group C (24 sessions for 8 weeks). The acupoints will be selected as Baihui (GV20), Yintang (GV29), Anmian (EX-HN22, bilaterally), Shenmen (HT7, bilaterally), Neiguan (PC6, bilaterally), and Sanyinjiao (SP6, bilaterally). The localization of the acupoints refers to the national standards of the People’s Republic of China issued by the State Bureau of Technical Supervision, “Nomenclature and location of acupuncture points” (GB/T12346-2006) and “Nomenclature and location of extra points in common use” (GB/T 40997–2021). The localization of the acupuncture points is shown in Figure 2.

The acupuncture needles used in this study are sterile, disposable acupuncture needles (Hwato, China). Optional sizes include 0.25 mm × 25 mm (1 inch) and 0.25 mm × 40 mm (1.5 inch).

Acupuncturists are licensed TCM practitioners with at least 3 years of clinical experience. The acupuncturist determines the acupuncture points on the skin, sterilizes them with 75% alcohol, and then penetrates the skin with the acupuncture needle. All points are treated with mild reinforcing and attenuating. After Deqi (the participant feeling of “numbness, soreness, heaviness, distention”), the needle is maintained for 5 s. Specific needling requirements: GV20 (horizontal needling 0.5–1 inch), GV29 (horizontal needling 0.3–0.5 inch), EX-HN22 (perpendicular needling 0.5–1 inch), HT7 (perpendicular needling 0.3–0.5 inch), SP6 (perpendicular needling 1–1.5 inch), PC6 (perpendicular needling 0.5–1 inch). The needle is retained for 30 min, and no manipulation is done during this period. Details of acupuncture treatment are shown in Table 1.

**Table 1:** Acupuncture treatment details based on the STRICTA 2010 checklist.

Item	Item number	Detail
1. Acupuncture rationale	(1a) Style of acupuncture (e.g., Traditional Chinese Medicine, Japanese, Korean, Western medical, Five Element, ear acupuncture, etc.)	Traditional Chinese Medicine (TCM)
	(1b) Reasoning for treatment provided, based on historical context, literature sources, and/or consensus methods, with references where appropriate	The treatment is based on traditional acupuncture theory, literature search, and expert’s consensus.
	(1c) Extent to which treatment was varied	Standardized acupuncture treatment
2. Details of needling	(2a) Number of needle insertions per subject per session (mean and range where relevant)	10	(2b) Names (or location if no standard name) of points used (uni/bilateral)	Baihui (GV20/uni), Yintang (GV29/uni), Anmian (EX-HN22/bilateral), Shenmen (HT7/bilateral), Sanyinjiao (SP6/bilateral), Neiguan (PC/bilateral)	(2c) Depth of insertion, based on a specified unit of measurement, or on a particular tissue level	From 10 to 40 mm	(2d) Response sought (e.g., de qi or muscle twitch response)	Deqi (numbness, soreness, heaviness, distention, etc.)	(2e) Needle stimulation (e.g., manual, electrical)	Manual acupuncture	(2f) Needle retention time	30 min	(2g) Needle type (diameter, length, and manufacturer or material)	Sterile, disposable acupuncture needles (diameter, 0.25 mm; length, 25/40 mm; Hwato, China).
3. Treatment regimen	(3a) Number of treatment sessions	12 ~ 24 sessions	(3b) Frequency and duration of treatment sessions	Three sessions a week (at least 1 day between treatments), for 4–8 weeks.
4. Other components of treatment	(4a) Details of other interventions administered to the acupuncture group (e.g., moxibustion, cupping, herbs, exercises, lifestyle advice)	sleep medications (periodic tracking); daily lifestyle advice	(4b) Setting and context of treatment, including instructions to practitioners, and information and explanations to patients	The trial will conducted at the Center of Preventive Medicine, Hospital of Chengdu University of TCM. Two researchers will diagnose and give informed consent to the participants, the acupuncturist will be responsible for providing treatment and lifestyle advice, and two designated researchers will be responsible for randomization and data collection. All participants of acupuncture group will be arranged to the acupuncture room for treatment.
5. Practitioner background	(5) Description of participating acupuncturists (qualification or professional affiliation, years in acupuncture practice, other relevant experience)	Trained, licensed acupuncturists with at least 2 years in acupuncture clinical practice
6. Control or comparator interventions	(6a) Rationale for the control or comparator in the context of the research question, with sources that justify this choice	The acupuncture regimen is the same for each group and based on traditional acupuncture theory, literature search and expert’s consensus.	(6b) Precise description of the control or comparator. If sham acupuncture or any other type of acupuncture-like control is used, provide details as for Items 1–3 above.	All three groups will receive the same real acupuncture treatment

### 3.7 Outcome measurement

Effective treatment should not only improve sleep parameters, but also produce clinically meaningful changes in daytime functioning, fatigue, anxiety, depression. Therefore, this trial will be evaluated from multiple perspectives to broaden the scope of outcome assessment (33).

#### 3.7.1 General information

(1) Participant demographics: gender, age, disease duration, educational level, occupation, sleeping environment, living environment, daily living situation, personality preference, income satisfaction, marital satisfaction, exercise habits.
(2) Vital signs: body temperature, heart rate, breathing, pulse, blood pressure, body mass index.

Baseline data are required to be completed within 48 h of randomization. Heart rate, pulse, and blood pressure will be assessed before and after each acupuncture treatment.

#### 3.7.2 Primary outcome

##### 3.7.2.1 ISI

ISI consists of seven items, with scores ranging from 0 to 4 for each item, and total scores ranging from 0 to 7 indicating normal sleep, from 8 to 14 indicating mild insomnia, from 15 to 21 indicating moderate insomnia, and from 22 to 28 indicating severe insomnia.

#### 3.7.3 Secondary outcomes

##### 3.7.3.1 PSQI

PSQI is used to rate the quality of a participant’s sleep in the previous month and consists of 18 items, comprising 7 components. This includes subjective sleep quality, sleep latency, sleep duration, habitual sleep efficiency, sleep disturbance, used sleep medication, and daytime dysfunction. Each component is scored from 0 to 3, and the cumulative score of each component is the PSQI total score. The total score ranges from 0 to 21, with higher scores indicating poorer sleep quality. Our prespecified primary endpoint is at the end of treatment and the secondary endpoint is 3 months after the end of treatment.

##### 3.7.3.2 SDS

SDS has a total of 20 items, each item is marked by 1–4 points, the total score is multiplied by 1.25 and rounded to an integer to get the standard score. The standard score normal upper limit reference value is 53 points; 53–62 points for mild depression, 63–72 points for moderate depression, and 72 points or more for severe depression.

##### 3.7.3.3 SAS

SAS has a total of 20 entries, each entry is marked by 1–4 points, the total score is multiplied by 1.25 and rounded to an integer to get the standard score. The boundary value of SAS standard score is 50 points; 50–59 points for mild anxiety, 60–69 points for moderate anxiety, and 69 points or more for severe anxiety.

##### 3.7.3.4 FSS

FSS has a total of 9 items, each scored on a scale of 1–7, with higher scores being associated with greater fatigue.

Primary and secondary outcomes will be evaluated at the following time points: Group 1 (0, 4 weeks), Group 2 (0, 4, 6 weeks), Group 3 (0, 4, 6, 8 weeks), and at follow-up (the first and third month after the end of treatment). The study schedule is depicted in Table 2.

**Table 2:** Study schedule.

Study period	Observation	Baseline	Treatment phase	Follow-up	
Timepoint	<7 days	0 week	4, 6, or 8 weeks	1 month	3 months
Enrollment					
Eligibility	×				
Informed consent		×			
Allocation		×			
Interventions					
Group 1			×		
Group 2			×		
Group 3			×		
Outcomes					
ISI		×	×	×	×
PSQI		×	×	×	×
SDS		×	×	×	×
SAS		×	×	×	×
FSS		×	×	×	×
Use of sleep medications			×	×	×
ISI < 8 points			×	×	×
Adverse events			×		

PSQI, Pittsburgh Sleep Quality Index; ISI, Insomnia Severity Index; SDS, Self-Rating Depression Scale; SAS, Self-Rating Anxiety Scale; FSS, Fatigue Severity Scale.

#### 3.7.4 Other outcomes

The percentage of participants with ISI < 8 points will be considered in our outcomes. During the observation period, in order to clarify whether acupuncture can reduce the use of sleep medication, we will record the type, dose, frequency, and duration of the participant’s daily sleep medication.

### 3.8 Safety assessments

We will record the possible adverse events in detail during the treatment period, including the time of occurrence, duration, and treatment measures of the adverse events. Common adverse events of acupuncture may include pain, bruising, and localized swelling at the site of acupuncture, and these possible conditions will disappear on their own after pressure or rest. Serious adverse events will be reported to the Ethics Committee of the Hospital of Chengdu, University of Traditional Chinese Medicine in a timely manner.

### 3.9 Quality control, data management, and monitoring

A detailed investigator’s manual has been developed prior to the start of the clinical trial, and all participating members of the study will be trained. Standard operation procedure (SOP) is strictly followed at every step of the process. Each participant has a unique case report form (CRF) for recording raw data, and all original CRFs are sealed by the project leader at the end of the trial. The designated investigator enters the raw data via a pre-designed, password-protected data management platform, and the data manager is blinded to verify the consistency of the original CRFs and the spreadsheets. An ombudsman (usually a panel of experts or an ethics committee) will intermittently monitor the acupuncturist and each step of the trial to protect the rights of the participants and ensure the quality of the study.

### 3.10 Statistical analysis

All statistical analysis will follow the intention-to-treat (ITT) principle, and the missing values will be supplemented by last observation carried forward (LOCF). For variables with normal distribution, one-way analysis of variance (ANOVA) will be used for comparison inter-group comparison, and paired samples *t*-test will be used for intra-group comparison. Kruskal-Wallis test will be used for non-normal distributed variables. Repeated measurements at different time points will be analyzed by using repeated-measures ANOVA. Data values will be mainly presented as the mean ± standard deviation or the median ± quartile spacing. While Chi-Square test/Fisher’s Exact test will be adopted to analyze categorical data. Categorical variables will be expressed as numbers and percentages. All statistical tests being bilateral at *p* < 0.05. Data analysis will be performed using IBM SPSS version 26.0 software (IBM Corp, New York).

## 4 Discussion

Although the pathophysiological characteristics of insomnia remain largely unknown, there is growing evidence that chronic insomnia has significant potential for increasing the risk of metabolic syndrome (34), hypertension (35) coronary heart disease (36), and is associated with maternal miscarriage, preterm birth, or perinatal depression (37). In addition, insomnia is an important risk factor for psychiatric disorders such as depression, anxiety, and alcohol dependence (38), and insufficient or poor-quality sleep can also lead to a decline in memory, attention, and responsiveness, resulting in cognitive impairments (39). Patients with insomnia have been reported to have a two-fold increased risk of depression (40) and more than a two-fold increased risk of dementia (41) compared to normal sleepers. The quality of life progressively deteriorates (42), directly or indirectly increasing mortality (43), and suicide rates (44).

Acupuncture treatment of CID is particularly important in the selection of acupoints. The acupoints prescriptions in this study are formulated based on the TCM theory, previous studies, and experts’ clinical experience, including GV20, GV29, EX-HN22, HT7, PC6, and SP6. TCM notes that insomnia is located in the heart and brain, and is closely related to the liver, spleen, and kidney. HT7 is the Shu-stream acupoint and Yuan-source acupoint of the Heart Meridian of Hand-Shaoyin; PC6 is the Luo-connecting point of the Pericardium Meridian of Hand-Shaoyin; and SP6 is the acupoint of the Spleen Meridian of Foot-Taiyin, which communicates with the liver, spleen, and kidney meridians. These three acupoints can play a role in calming the heart, tranquilizing the mind, and nourishing the blood. GV20 is the Crossing acupoint of the Governor Meridian, the Bladder Meridian of Foot-Taiyang, the Gallbladder Meridian of Foot-Shaoyang, the Triple Energizer Meridian of Hand-Shaoyang, and the Liver Meridian of Foot-Jueyin, located at the top of the head, with a close relationship to the brain, able to awaken consciousness and open the cardiac orifice, tranquilizing and sedating the mind. GV29 and EX-HN22 are both extra-meridian points that are experienced in treating insomnia. Studies have shown that acupuncture points such as HT7, SP6, and GV20 can regulate neurotransmitters such as 5-hydroxytryptamine, norepinephrine, dopamine, γ-aminobutyric acid, glutamate, acetylcholine, and orexin, to adjust the structure of sleep and improve the state of anxiety and depression (45–47). Therefore, this study formulated acupoint prescription on these bases.

Acupuncture dose-effect relationships, such as the depth of acupuncture, deqi and arrival of qi, acupuncture sessions, the time of retaining the needle, the manipulation of acupuncture, the interval time between acupuncture, and the duration of treatment, have been documented as early as the Huangdi’s Internal Classics. However, the rapid development of the concept of evidence-based medicine has brought a series of challenges to acupuncture (48). Given the specificity of acupuncture therapy, quantifying the effect of acupuncture has become a key element of current acupuncture research. At present, there are fewer reports in the literature on the dose-effect relationship of acupuncture in CID, and some existing clinical studies have observed the improvement of sleep by acupuncture from different amounts of manipulative stimulation (49, 50). But the manipulation of acupuncture is complicated and difficult to completely quantify, even though some instruments simulate acupuncture manipulation, it is still not possible to realize the needle sensation, regulate the qi, and reinforce and reduce methods (51).

CID, as a common and highly prevalent condition, lacks direct clinical evidence of the cumulative effect of acupuncture. With the popularization and expansion of medical insurance, more patients with CID are in a position to complete the entire acupuncture course, but it is not clear whether different acupuncture courses produce significant differences in clinical efficacy. Therefore, this study takes the acupuncture treatment course as an entry point to assess the clinical efficacy of different acupuncture courses for CID, under the premise of relative quantification of acupuncture manipulation and standardization of frequency and acupoints, which is conducive to narrowing the differences in health economics and is of great significance in guiding clinical decision-making and carrying out further scientific research.

### 4.1 Advantages and limitations

This is a head-to-head comparative effectiveness trial. To our knowledge, this study is the first to directly observe the dose-effect relationship of acupuncture for CID using different acupuncture courses as a starting point. Second, we are not restricting participants from using sleep medications. During the study period, we will follow participants’ use of psychotropic medications (e.g., antidepressants, anxiolytics) and/or sleep medications. In addition, in order to minimize the bias caused by the details of acupuncture, we unified the acupuncture protocol, including the number of needle insertions, depth of insertion, needle type, response sought, needle frequency, and retention time.

However, there are some limitations of the study. First, the uniformity of the acupuncture protocol also meant that the points are not to be dialectically selected and individual differences will not be considered, which may underestimate the overall efficacy of acupuncture. Second, due to the specificity of the study design and acupuncture, we are unable to blind patients and acupuncturists. The researchers responsible for data collection, statistical analysis, and efficacy evaluation are unaware of the groupings to minimize bias in reporting subjective results. In addition, the results of this study are subjective, and we do not use aids for the objective assessment of sleep, such as polysomnography and the Mean Sleep Latency Test (MSLT), which are not applicable to the routine assessment of CID with poor patient compliance (11).

The scope of the dose-effect relationship of acupuncture is very wide. In this trial, one of the factors that may have a strong influence on the efficacy (different treatment courses) will be selected for comparative study. In future studies, under the premise of relative quantification of manipulation, different number of sessions per week, continuous treatment or interval treatment, the choice of unilateral or bilateral acupoints, and needle retention time will all be the focus of research on the dose-effect relationship of acupuncture for CID. At the same time, we prefer to assess clinical efficacy by a combination of subjective and objective indicators if we can obtain consent from a large number of participants.

## Ethics statement

The Ethics Committee of Hospital of Chengdu University of Traditional Chinese Medicine has approved the study protocol (Ethical approval number: 2023KL-064). Written informed consent will be obtained from participants.

## Author contributions

FZ: Conceptualization, Methodology, Writing – original draft, Writing – review & editing. JL: Writing – original draft. YW: Writing – review & editing. TM: Writing – original draft, Writing – review & editing. TW: Writing – original draft. BY: Writing – original draft. RM: Data curation, Methodology, Supervision, Writing – review & editing. JW: Supervision, Writing – original draft, Writing – review & editing.
